# Pain Modulation in WAG/Rij Epileptic Rats (A Genetic Model of Absence Epilepsy): Effects of Biological and Pharmacological Histone Deacetylase Inhibitors

**DOI:** 10.3389/fphar.2020.549191

**Published:** 2020-12-03

**Authors:** Carmen De Caro, Lorenzo Di Cesare Mannelli, Jacopo Junio Valerio Branca, Laura Micheli, Rita Citraro, Emilio Russo, Giovambattista De Sarro, Carla Ghelardini, Antonio Calignano, Roberto Russo

**Affiliations:** ^1^ Department of Pharmacy, University of Naples Federico II, Naples, Italy; ^2^ Department of Science of Health, School of Medicine and Surgery, University of Catanzaro, Catanzaro, Italy; ^3^ Department of Neuroscience, Psychology, Drug Research and Child Health–Neurofarba–Section of Pharmacology and Toxicology, University of Florence, Florence, Italy; ^4^ Department of Experimental and Clinical Medicine, Anatomy and Histology Section, University of Florence, Florence, Italy

**Keywords:** pain, epilepsy, WAG/Rij rats, histone deacetylase-inhibitors, sodium butyrate, valproic acid, hyperalgesia, allodynia

## Abstract

Epigenetic mechanisms are involved in epilepsy and chronic pain development. About that, we studied the effects of the natural histone deacetylase (HDAC) inhibitor sodium butyrate (BUT) in comparison with valproic acid (VPA) in a validated genetic model of generalized absence epilepsy and epileptogenesis. WAG/Rij rats were treated with BUT (30 mg/kg), VPA (300 mg/kg), and their combination (BUT + VPA) daily *per os* for 6 months. Rats were subjected at Randall–Selitto, von Frey, hot plate, and tail flick tests after 1, 3, and 6 months of treatment to evaluate hypersensitivity to noxious and non-noxiuous stimuli. Moreover, PPAR-**γ** (G3335 1 mg/kg), GABA-B (CGP35348 80 mg/kg), and opioid (naloxone 1 mg/kg) receptor antagonists were administrated to investigate the possible mechanisms involved in analgesic activity. The expression of NFkB, glutathione reductase, and protein oxidation (carbonylation) was also evaluated by Western blot analysis. WAG/Rij rats showed an altered pain threshold throughout the study (*p* < 0.001). BUT and BUT + VPA treatment reduced hypersensitivity (*p* < 0.01). VPA was significantly effective only after 1 month (*p* < 0.01). All the three receptors are involved in BUT + VPA effects (*p* < 0.001). BUT and BUT + VPA decreased the expression of NFkB and enhanced glutathione reductase (*p* < 0.01); protein oxidation (carbonylation) was reduced (*p* < 0.01). No effect was reported with VPA. In conclusion BUT, alone or in coadministration with VPA, is a valuable candidate for managing the epilepsy-related persistent pain.

## Introduction

Chronic pain involves the central nervous system (CNS) independently from the origin of damage. A maladaptive plasticity of spinal cord and brain leads to molecular, cellular, and electrophysiological alterations that induce persistence pain (Latremoliere and Woolf, 2009). Hyperexcitability of nervous cells is a common feature for both epilepsy and chronic pain. Therefore, pain syndromes are common comorbid conditions in patients with epilepsy (Bianchin et al., 2010; Ottman et al., 2011). This sensitized, overstimulated system, characteristic of chronic pain usually responds to antiepileptic drugs (Gilron et al., 2015). Gabapentin or pregabalin is recommended as first line, and several other antiepileptics like lamotrigine, oxcarbazepine, topiramate, VPA are effective in subgroups of patients affected by neuropathic pain (Bouhassira and Attal, 2018). Indeed, the normalization of ion channels functioning and neurotransmitter’s signaling leads to pain relief. In WAG/Rij rats, a consolidated genetic model of generalized absence epilepsy, which appears after the first month of life, and epileptogenesis (Russo E. et al., 2016), the hypersensitivity to thermal noxious stimulus both in the interictal and ictal periods, was described (Velioglu et al., 2017; Velioglu et al., 2018). The study of pain sensitivity in epileptic subjects may offer important information for the comprehension of both pathologies, elucidating neural circuits and suggesting possible novel therapeutic approaches.

In this view, BUT is an intriguing candidate. BUT is a short-chain fatty acid present in food and produced endogenously by commensal anaerobic fermentation of undigested carbohydrates in the colon; it is currently considered an active postbiotic (Topping and Clifton, 2001; Stilling et al., 2016). BUT was able to reduce acute and chronic animal pain models. These effects lead to the activation of the peroxisome proliferator-activated receptors (PPARs) (Russo R. et al., 2016). Moreover, other CNS disorders are responsive to BUT treatment (Stilling et al., 2016). In a model of cognitive dysfunction, BUT improved memory in parallel to the restoration of physiological levels of glial derived neurotrophic factor (GDNF) and brain derived neurotrophic factor (BDNF) (Barichello et al., 2015). Similarly, BUT increased neurotrophic factors levels in hippocampus and frontal cortex of ouabain-treated rats significantly reducing maniac-like behavior (Valvassori et al., 2016). The neuroprotective effects of BUT were also highlighted in ischemic stroke and neurodegenerative conditions (Rotilio et al., 2004). BUT’s efficacy against different alteration across the CNS may be justified by its property to remodel histones inhibiting histone deacetylase (HDAC) (Russo et al., 2018). Epigenetics, the process by which gene activity is altered without alterations in the DNA sequence, is thought to contribute to epilepsy and epileptogenesis, and HDAC inhibitors like VPA are currently used in therapy with significant results in terms of neuroprotection (Citraro et al., 2018; Romoli et al., 2019).

Hypothesizing a direct link between pain and absence seizure, in the present work we aim to 1) investigate the pain threshold of WAG/Rij rats during the first 7 months of animal’s life and 2) evaluate the pain-relieving effects induced by repeated administration of BUT in comparison to those evoked by VPA, a very well-known epigenetic drug also able to control pain perception, or by the association between BUT and VPA.

## Materials and Methods

### Animals

Male Wistar and WAG/Rij rats (4 weeks of age), originally purchased from Charles River Laboratories s.r.l. (Calco, Lecco, Italy), were housed three per cage and kept under controlled environmental conditions (60 ± 5% humidity; 22 ± 2°C; 12/12 h reversed light/dark cycle); food and water were available *ad libitum* throughout the study. All behavioral tests were performed between 9:00 AM and 5:00 PM. Animal care and manipulations were conducted in conformity with international and national law and policies (EU Directive 2010/63/EU for animal experiments, ARRIVE guidelines, and the Basel declaration including the 3R concept). The procedure reported here was approved by the Institutional Committee on the Ethics of Animal Experiments (CVS) of the University of Naples Federico II and by Ministero della Salute (protocol n. 371/2017-PR, February 20, 2017).

### Experimental Protocol

In 1-month-old male WAG/Rij rats (*n* = 28), BUT (30 mg/kg/day; p.o.), VPA (300 mg/kg/day; p.o.), and their coadministration (p.o., 30 and 300 mg/kg/day, respectively) were daily administered for 6 months. Drugs were solubilized in tap water and administrated by bottle, as previously described (Citraro et al., 2020. Behavioral tests were performed on months 1, 3, and 6 of treatment. Dosages of BUT and VPA were chosen on the bases of previously published data (Russo R. et al., 2016; Citraro et al., 2020). Rechallenge and pharmacodynamic studies were performed on month 7 after a 30-day period free of substances. After the washout period the respective groups of animals were treated daily p.o. for 1 week with BUT (30 mg/kg), VPA (300 mg/kg), and BUT + VPA (30 + 300 mg/kg), respectively. The selective antagonists G3335 (1 mg/kg), CGP35348 (80 mg/kg), and naloxone (1 mg/kg) were administered intraperitoneally 30 min before the tests. At these doses, antagonists were not able to modify *per se* the pain threshold. All compounds were purchased from Sigma-Aldrich (Italy).

### Von Frey Test

The animals were placed in 20 cm × 20 cm Plexiglas boxes equipped with a metallic meshy floor, 20 cm above the bench. A habituation of 30 min was allowed before the test. An electronic Von Frey hair unit (Ugo Basile, Varese, Italy) was used: the withdrawal threshold was evaluated by applying force ranging from 0 to 50 g with a 0.2 g accuracy. Punctuate stimulus was delivered to the mid-plantar area of each anterior paw from below the meshy floor through a plastic tip, and the withdrawal threshold was automatically displayed on the screen. Paw sensitivity threshold was defined as the minimum pressure required to elicit a robust and immediate withdrawal reflex of the paw. Voluntary movements associated with locomotion were not taken as a withdrawal response. Stimuli were applied on each anterior paw with an interval of 5 s. The measure was repeated five times and the final value was obtained by averaging the five measures. The data were collected by an observer who was blinded to the protocol (Sakurai et al., 2009; Di Cesare Mannelli et al., 2012).

### Randall–Selitto Test

Mechanical hypersensitivity to a noxious stimulus was measured using an analgesimeter (Ugo Basile, Varese, Italy). Briefly, a constantly increasing pressure was applied to a small area of the dorsal surface of the hind paw using a blunt conical mechanical probe. Mechanical pressure was increased until vocalization or a withdrawal reflex occurred while rats were lightly restrained. Vocalization or withdrawal reflex thresholds were expressed in grams. These limits assured a more precise determination of mechanical withdrawal threshold in experiments aimed to determine the effect of treatments. Hyperalgesia was assessed on both paws at 1, 3, and 6 months of treatment. Each paw was tested one per session. An arbitrary cut-off value of 250 g was adopted. The data were collected by an observer who was blinded to the protocol (Leighton et al., 1988).

### Hot Plate Test

The hot plate test was used to evaluate the response to a noxious thermal stimulus. During the experiment, rats were introduced into an open-ended cylindrical space with a floor consisting of a heated plate. The plate heated to a constant temperature (55 ± 1°C) produces two behavioral components that can be measured in terms of their reaction times (s), namely paw licking and jumping. Both are considered to be supraspinally integrated responses. The cut-off imposed was 30 s to avoid tissue damage. The reaction time was subsequently assessed 1–6 months after chronic oral treatments (Doncheva et al., 2019). The data were collected by an observer who was blinded to the protocol.

### Tail Flick Test

Tail flick test is an extensively used test of nociception in rats. The nociceptive assay is based on the measurement of the latency of the avoidance response to thermal noxious stimulus in rodents. The tail-flick was evoked by a source of radiant heat (infrared, 60 IR was set with 20 s ramp time) which was focused on the dorsal surface of the tail, and the time it takes until the animal flicks the tail away from the beam is measured. The cut-off imposed was 20 s to prevent tissue damages (Mondal et al., 2019). The data were collected by an observer who was blinded to the protocol.

### Western Blot Analysis

The lumbar portions (L4–L6) of the spinal cord of rats from the different experimental groups were collected on month 6 after behavioral measurements. Tissue was suspended in lysis buffer and sonicated on ice using three 10-s bursts at high intensity with a 10-s cooling period between each burst. After centrifugation (13,000 × g for 15 min at 4°C) aliquots containing 20 μg total protein were separated on a 4–12% sodium dodecyl sulfate (SDS)-polyacrylamide gel by electrophoresis and transferred onto nitrocellulose membranes (Bio-Rad, Italy). Specific antisera against NFkB (1:1,000; Novus Biologicals, CO, United States), SOD1 antiserum (1:1,000; Santa Cruz Biotechnology, CA, United States), SOD2 (1:1,000; Santa Cruz Biotechnology, CA, United States), glutathione reductase antiserum (1:500; Abcam, Cambridge, United Kingdom), phophoERK1,2 (1:1,000; Abcam, Cambridge, United Kingdom) and BAX (1:1,000; Santa Cruz Biotechnology, CA, United States) were used to assess the analysis.

Densitometric analysis was performed using the “ImageJ” analysis software (NIH Bethesda, Maryland, United States), and results were normalized to β-actin, α-tubulin, and GAPDH immunoreactivity (1:2,000 rat antiserum, Cell Signaling, United States) as internal control.

### Carbonylated Proteins

Protein extracts from the lumbar portion of the spinal cord samples were prepared and separated as described above. Membranes were blocked with 5% non fat dry milk in phosphate-buffered saline (PBS) containing 0.1% Tween 20 (PBST) and then probed overnight with primary antibody specific vs DNPH (Sigma-Aldrich, Italy) 1:5,000 in PBST/5% non-fat dry milk. After washing with PBST, the membranes were incubated for 1 h in PBST containing the appropriate horseradish peroxidase-conjugated secondary antibody (1:5,000; Cell Signaling, United States) and again washed. ECL (Pierce, United States) was used to visualize the peroxidase-coated bands. Densitometric analysis was performed using the “ImageJ” software (NIH Bethesda, Maryland, United States). For each experiment, the density of all bands shown in a lane was reported as mean. β-Actin (1:2,000 rat antiserum, Cell Signaling, United States) normalization was performed for all samples (Di Cesare Mannelli et al., 2012).

### Statistical Analysis

Measurements were performed on at least six animals for each group analyzed in two different experimental sets by researchers blinded to the treatment procedure. Results were expressed as means ± S.E.M. and the significance of differences among groups was determined by one- or two-way analysis of variance ANOVA test. A Bonferroni’s significant difference procedure was used as a post hoc comparison. *p* values less than 0.05 were considered significant. Data were analyzed using the Origin 8.1 software (OriginLab, Northampton, MA, United States).

## Results

### Difference Between WAG/Rij and Wistar Rats in Pain Perception

**FIGURE 1:**
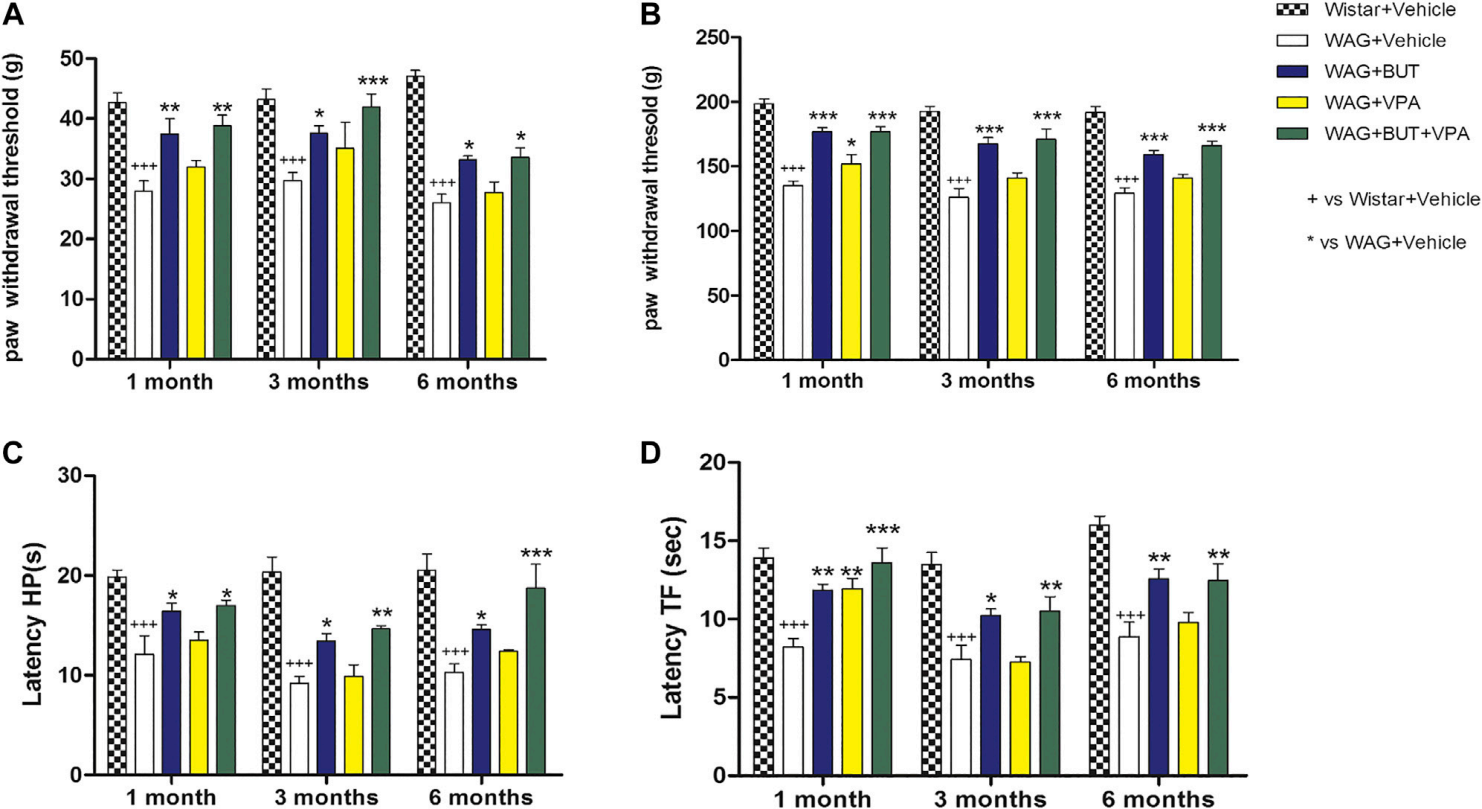
Pain threshold measurements. WAG/Rij animals were treated daily p.o. from day 1 with BUT (30 mg/kg), VPA (300 mg/kg), (BUT + VPA, 30 + 300 mg/kg) or vehicle. Wistar rats received vehicles. The response to a non-noxious mechanical stimulus was measured by the von Frey test **(A)**, the response to a noxious mechanical stimulus was measured by the Randall-Selitto test **(B)**, the response to a noxious thermal stimuli was measured by the hot plate test **(C)** and by the tail flick test **(D)**. The tests were performed after 1, 3, and 6 months. Data are expressed as mean ± S.E.M. of at least six rats per group. Statistical analysis is two-way ANOVA followed by Bonferroni’s *post hoc* comparison. ^+++^ *p* < 0.001 vs Wistar + Vehicle; *p < 0.05 ** < 0.01 and ***p < 0.001 vs WAG + vehicle.

The pain threshold of WAG/Rij rats was measured at different ages in comparison to Wistar rats considered as nonpathological control (neither epileptic nor with an altered pain threshold) (Velioglu et al., 2017). The first evaluation performed after 1 month of treatment (2 months of age) revealed that WAG/Rij-vehicle group was significantly hypersensitive to a non-noxious mechanical stimulus compared to Wistar-vehicle animals (Figure 1A; pain threshold decrease = −33%, Von Frey test ^+++^ *p* < 0.001 vs Wistar + vehicle; ^*^ *p* < 0.05 and ***p < 0.001 vs WAG + vehicle). This response, related to the clinical sign of allodynia, was maintained at 3 and 6 months; no difference was evidenced between WAG/Rij rats of the different ages tested.

As shown in Figure 1B, the alteration of pain threshold was observed also in response to a noxious mechanical stimulus (pain threshold decrease = −30%; Randall–Selitto test ^+++^ *p* < 0.001 vs Wistar + vehicle; ^*^ *p* < 0.05; and ***p < 0.001 vs WAG + vehicle), and WAG/Rij presented a stable hyperalgesia-like condition at 1, 3, and 6 months with respect to Wistar rats; again, no difference was observed between the various ages considered. The development of hypersensitivity was higher by using thermal noxious stimuli as in the hot plate and tail flick tests, pain threshold decreased by 50% and 43%, respectively, in comparison to Wistar rats (Figures 1C,D; ^+++^ *p* < 0.001 vs Wistar + vehicle; ^*^ *p* < 0.05, **p < 0.01; and ***p < 0.001 vs WAG + vehicle); also, this parameter was constantly maintained over aging in WAG/Rij rats. Considering age as a factor, also in Wistar rats, no difference was observed for any test at any age considered.

### Effect of Butyrate and Valproic Acid Treatments in WAG/Rij Rats Pain Perception

**FIGURE 2:**
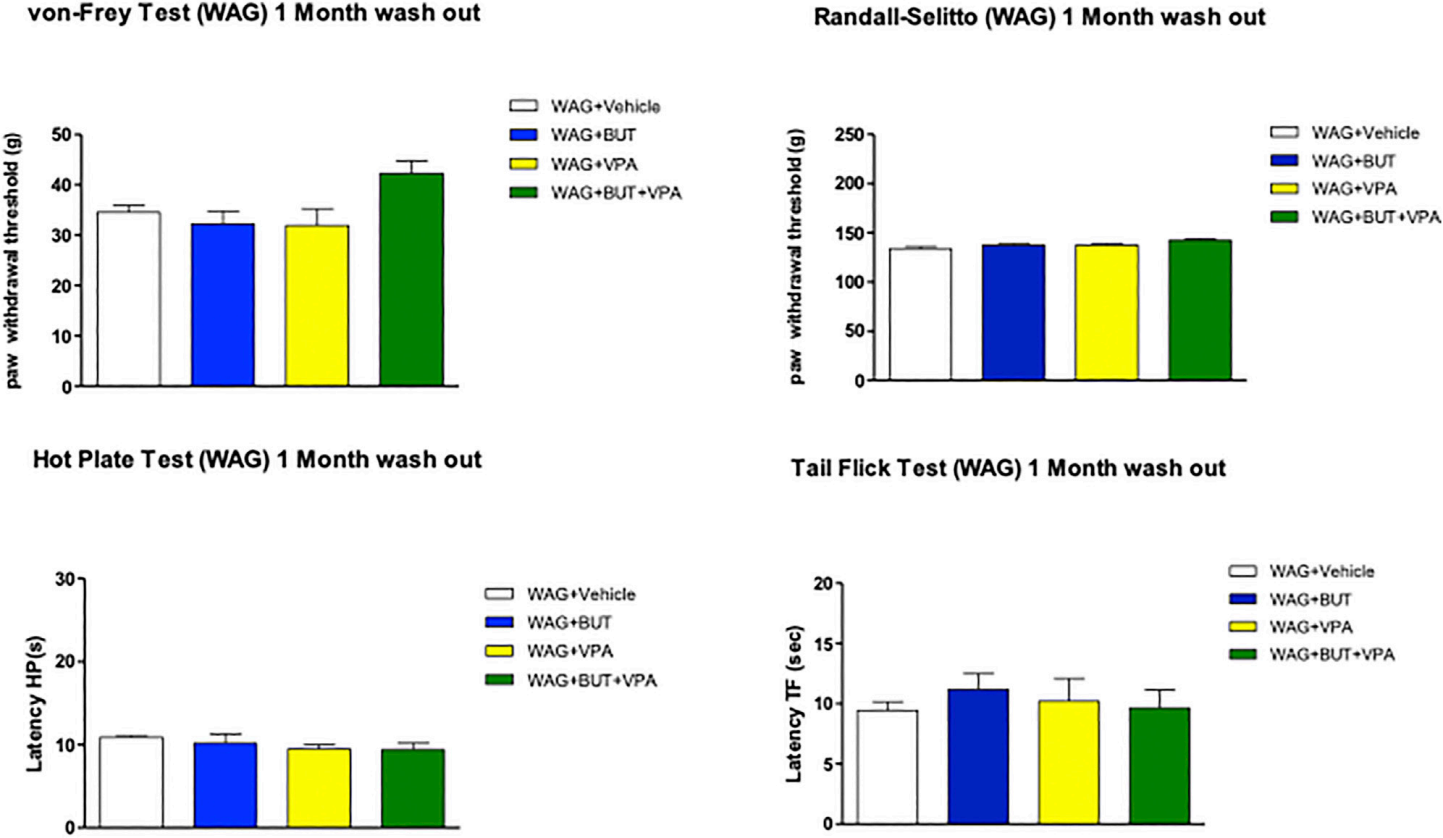
Pain threshold after 1 month of wash out following 6 months of treatments. WAG/Rij animals were treated daily p.o. from day 1 with BUT (30 mg/kg), VPA (300 mg/kg), (BUT + VPA, 30 + 300 mg/kg) or vehicle for 6 months. Thereafter all treatments were suspended for 1 month, pain threshold was measured by von Frey, Randall–Selitto, hot plate, and tail flick tests. Data are expressed as mean ± S.E.M. of at least six rats per group. Statistical analysis is one-way ANOVA followed by Bonferroni’s post hoc comparison.

**FIGURE 3:**
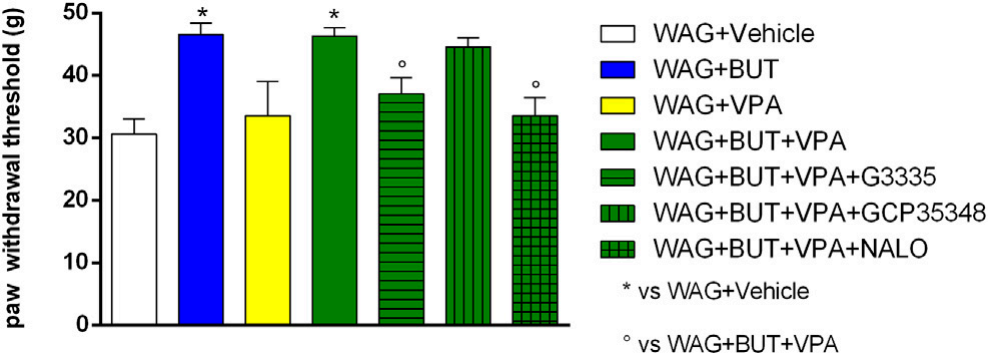
Effect of specific antagonists. Pain threshold, response to a non-noxious mechanical stimulus was measured. In von Frey test, WAG/Rij rats were treated daily p.o. for 1 week with BUT (30 mg/kg), VPA (300 mg/kg), (BUT + VPA, 30 + 300 mg/kg). On day 8, the von Frey test was performed. Separately, the PPAR-**γ** antagonist G3335 (1 mg/kg), the GABA-B antagonist CGP35348 (80 mg/kg) and the opioid antagonist naloxone (NALO 1 mg/kg) were administered intraperitoneally 30 min before the tests to the BUT + VPA group. Data are expressed as mean ± S.E.M. of at least six rats per group. Statistical analysis is one-way ANOVA followed by Bonferroni’s *post hoc* comparison. *p < 0.05 vs WAG + vehicle; °*p* < 0.05 vs WAG + BUT + VPA.

**FIGURE 4:**
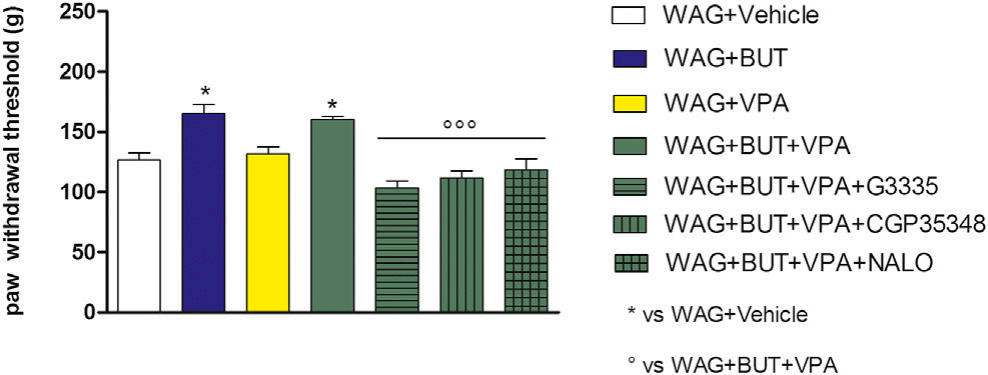
Effect of specific antagonists. Pain threshold, response to a noxious mechanical stimulus was measured. WAG/Rij rats were treated daily p.o. for 1 week with BUT (30 mg/kg), VPA (300 mg/kg), (BUT + VPA, 30 + 300 mg/kg). On day 8, the Randall–Selitto test was performed. Separately, the PPAR-**γ** antagonist G3335 (1 mg/kg), the GABA-B antagonist CGP35348 (80 mg/kg) and the opioid antagonist naloxone (NALO 1 mg/kg) were administered intraperitoneally 30 min before the tests to the BUT + VPA group. Data are expressed as mean ± S.E.M. of at least six rats per group. Statistical analysis is one-way ANOVA followed by Bonferroni’s *post hoc* comparison.*p < 0.05 vs WAG + vehicle; ^ºº^ *p* < 0.001 vs WAG + BUT + VPA.

With the aim of counteracting WAG/Rij rats’ hypersensitivity, different, repeated treatments were performed on separate groups of animals. Valproic acid (VPA; 300 mg/kg), sodium butyrate (BUT; 30 mg/kg), and the combination of both (BUT + VPA) were orally administered daily. After 1 month of treatment and throughout the period (3 and 6 months), BUT significantly normalized pain perception of WAG/Rij rats. In all performed tests, BUT-treated animals showed lower hyperalgesia- and allodynia-like sensitivity. Pain threshold was enhanced in response to mechanical and thermal insults (Figures 1–4). VPA treatment was only significantly effective after 1 month in the Randall–Selitto and tail flick tests (mechanical and thermal hyperalgesia; Figures 1, 4) but not in all other tests; furthermore, VPA effects disappeared during treatment with the drug being ineffective at 3 and 6 months. The combination (BUT + VPA) was effective in all tests after 1, 3, and 6 months of treatment in WAG/Rij rats. Pain relieving effect of BUT + VPA was always higher than that of VPA alone; while only in some measurements of thermal sensitivity (hot plate at 6 months; tail flick at 1 month) BUT + VPA was significantly more effective than BUT alone allowing to reach a complete normalization of pain threshold (Figures 1A–D; ^+++^ *p* < 0.001 vs Wistar + vehicle; ^*^ *p* < 0.05, **p < 0.01; and ***p < 0.001 vs WAG + vehicle). After 6 months, treatments were suspended to allow a period of washout. One month after, pain threshold was assessed in the different groups; in Figure 2 is shown that there were not significant pain relieving effects with exception of BUT + VPA in the Randall–Selitto test. Hypersensitivity of WAG/Rij rats in comparison to Wistar rats was similar to that observed at 6 months (data not shown).

### Rechallenge and Pharmacodynamic Investigation

**FIGURE 5:**
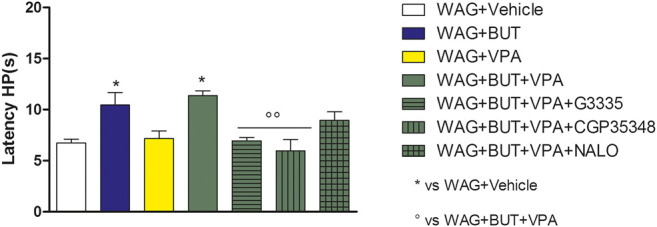
Effect of specific antagonists. Pain threshold, response to a noxious thermal stimulus was measured. WAG/Rij rats were treated daily p.o. for 1 week with BUT (30 mg/kg), VPA (300 mg/kg), (BUT + VPA, 30 + 300 mg/kg). On day 8, the hot plate test was performed. Separately, the PPAR-**γ** antagonist G3335 (1 mg/kg), the GABA-B antagonist CGP35348 (80 mg/kg) and the opioid antagonist naloxone (NALO 1 mg/kg) were administered intraperitoneally 30 min before the tests to the BUT + VPA group. Data are expressed as mean ± S.E.M. of at least six rats per group. Statistical analysis is one-way ANOVA followed by Bonferroni’s *post hoc* comparison. *p < 0.05 vs WAG + vehicle; ^ººº^ *p* < 0.01 vs WAG + BUT + VPA.

**FIGURE 6:**
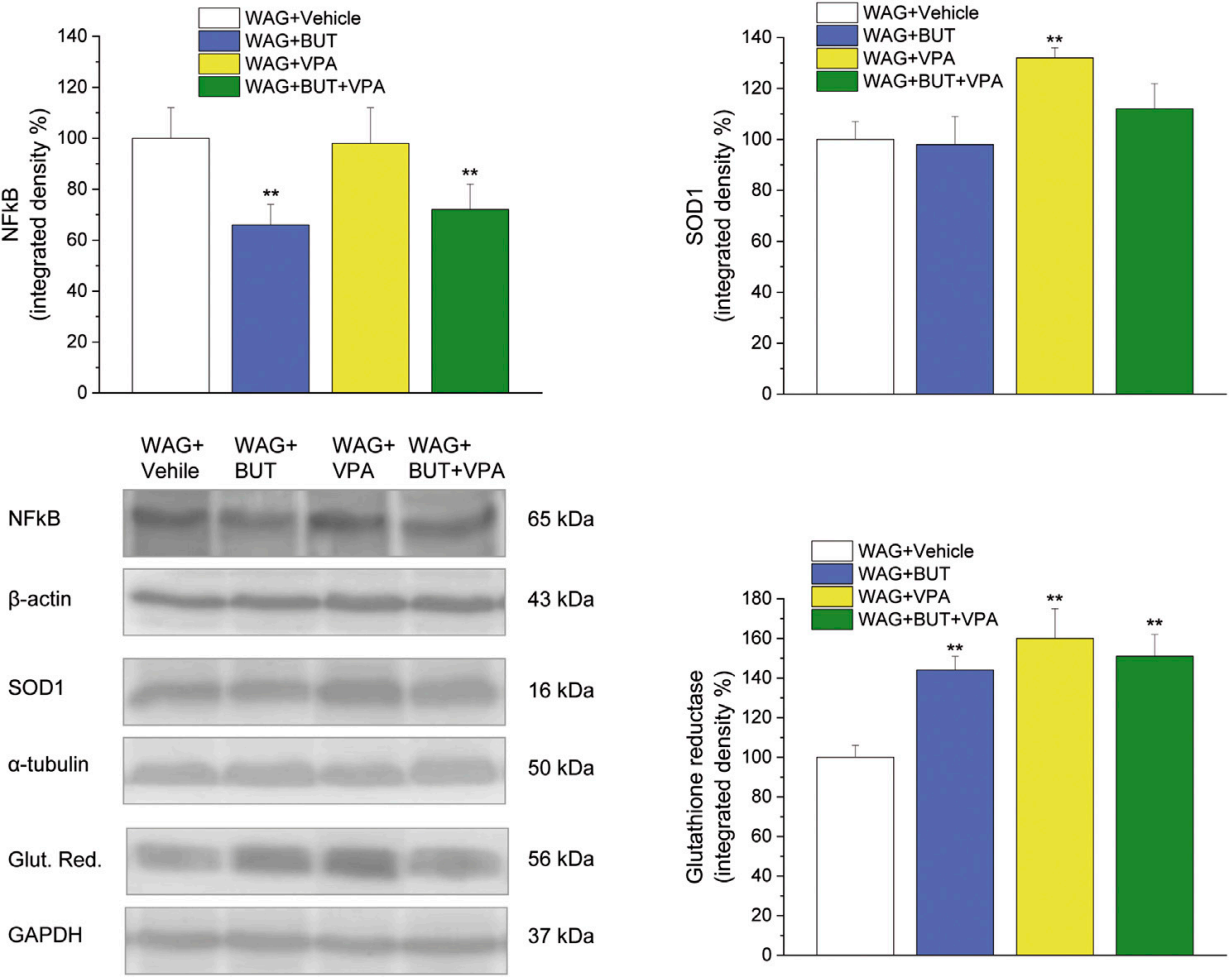
Spinal cord, molecular analysis. NFkB, SOD1 and glutathione reductase were measured in the spinal cord of WAG/Rij rats treated daily p.o. from day 1 with BUT (30 mg/kg), VPA (300 mg/kg), (BUT + VPA, 30 + 300 mg/kg) or vehicle for 6 months. Densitometric analysis and representative Western blot are shown. β-Actin, α-tubulin, GAPDH normalization were performed for NFkB, SOD1 and glutathione reductase, respectively. Data are expressed as mean ± S.E.M. of at least six rats per group. Statistical analysis is one-way ANOVA followed by Bonferroni’s *post hoc* comparison. **p < 0.01 vs WAG + vehicle.

**FIGURE 7:**
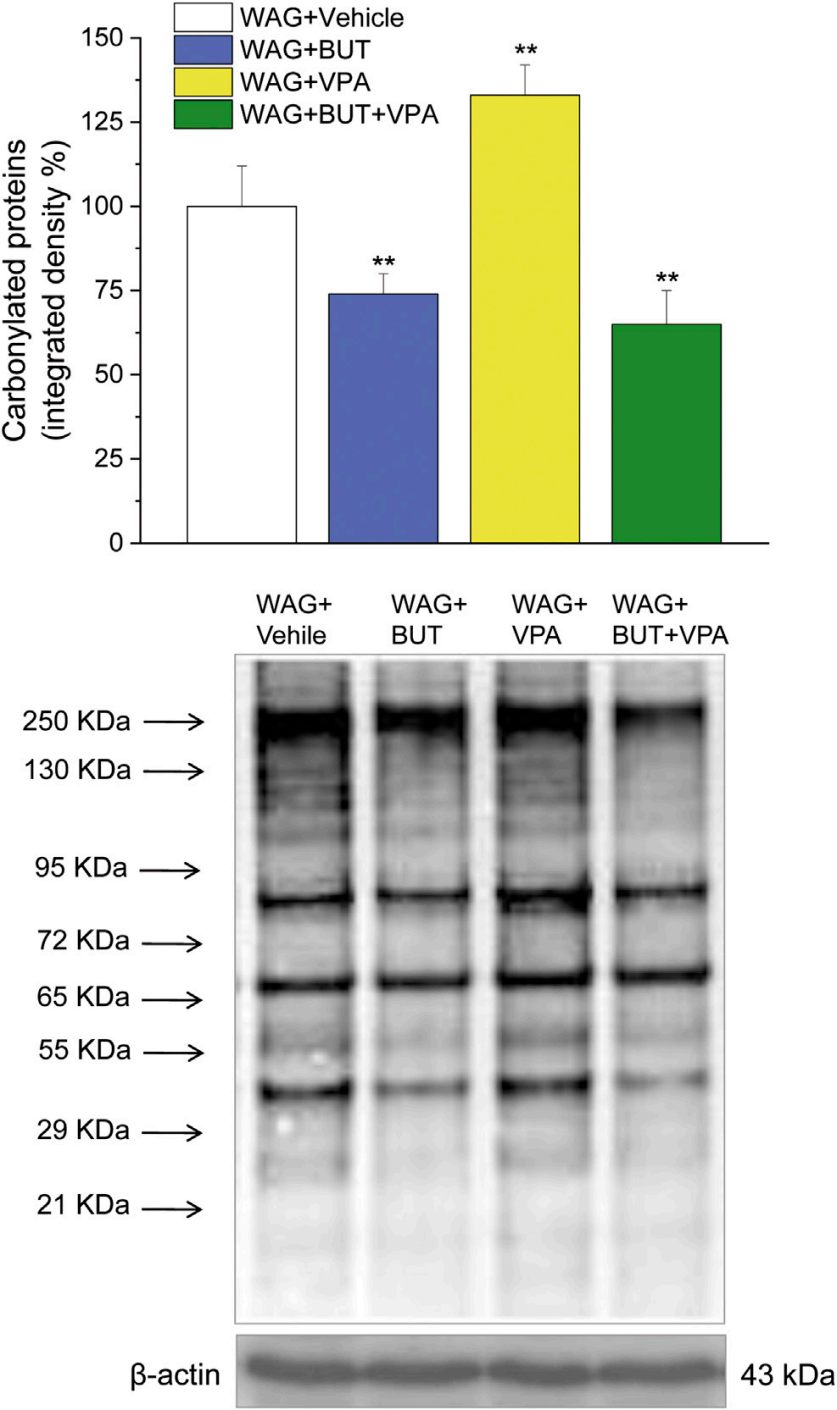
Spinal cord, oxidation. Carbonylated proteins were measured in the spinal cord of WAG/Rij rats treated daily p.o. from day 1 with BUT (30 mg/kg), VPA (300 mg/kg), (BUT + VPA, 30 + 300 mg/kg) or vehicle for 6 months. Densitometric analysis (top panel) and representative Western blot (lower panel) are shown. β-Actin normalization was performed for each sample. Data are expressed as mean ± S.E.M. of at least six rats per group. Statistical analysis is one-way ANOVA followed by Bonferroni’s *post hoc* comparison. **p < 0.01 vs WAG + vehicle.

To evaluate the drugs’ responsivity of animals after 1 month washout, the respective groups of animals were treated daily p.o. for 1 week with BUT (30 mg/kg), VPA (300 mg/kg), and BUT + VPA (30 + 300 mg/kg), respectively. On day 8, treatments were able to modify the pain threshold similarly to that observed on month 6 (Figures 3–5). BUT and the coadministration of BUT + VPA rapidly relieved WAG/Rij-related hypersensitivity to obtain the same results collected before the washout. VPA was ineffective. To go deep inside the pharmacodynamic mechanisms, pain-relevant receptor signals were analyzed by the use of specific antagonists. In particular, to test the role of PPAR-**γ**, GABA-B, and opioid receptors in the pain-relieving effect shown by the association between BUT and VPA, the selective antagonists G3335 (1 mg/kg), CGP35348 (80 mg/kg), and naloxone (1 mg/kg) were intraperitoneally injected 30 min before the tests. Figure 3 displays the results of the von Frey test; the efficacy of BUT + VPA in reducing hypersensitivity to a non-noxious mechanical stimulus was significantly reduced by pharmacologically blocking PPAR-**γ** and opioid receptors but not GABA-B receptors (^*^ *p* < 0.05 vs WAG + vehicle; ^°^ *p* < 0.05 vs WAG + BUT + VPA). As regards the response to noxious stimuli, the antihypersensitivity effects to a mechanical stress (Randall–Selitto test) were reduced by all antagonists (Figure 4; ^*^ *p* < 0.05 vs WAG + vehicle; ^ººº^ *p* < 0.001 vs WAG + BUT + VPA), whereas the effects against a thermal stimulus (hot plate test) were significantly reduced by PPAR-**γ** and GABA-B antagonists (Figure 5; ^*^ *p* < 0.05 vs WAG + vehicle; ^ºº^ *p* < 0.01 vs WAG + BUT + VPA). *Ex vivo*, the spinal cord (as the first station of pain signaling in the central nervous system) was analyzed measuring the protein levels of pain-related markers of damage or protection. Six months of treatment with BUT significantly reduced the expression of the inflammatory factor NFkB; the same effect was obtained also in animals that received the coadministration of BUT and VPA. On the contrary, VPA alone did not induce any effect (Figure 6; **p < 0.01 vs WAG + vehicle). The detoxifying enzyme SOD1 was significantly enhanced by VPA. Glutathione reductase, essential for the glutathione redox cycle, was significantly increased by BUT, VPA, and BUT + VPA to a similar extent (Figure 6; **p < 0.01 vs WAG + vehicle). Accordingly, the final oxidative state, evaluated as quantity of carbonylated proteins, was significantly improved by BUT and BUT + VPA; on the contrary, VPA treatment increased the concentration of carbonylated proteins in comparison to WAG/Rij treated with vehicle (Figure 7; **p < 0.01 vs WAG + vehicle).

## Discussion

Considering our initial hypothesis, our study indicates that absence seizures and pain threshold are linked in the WAG/Rij rats sharing common mechanisms even though the number of seizures is not apparently linked to pain itself, as in the case of other comorbidities in this model (Sarkisova et al., 2010; Russo and Citraro, 2018; Leo et al., 2019); furthermore, we demonstrate the pain relieving efficacy of BUT and BUT + VPA in WAG/Rij rats. Genetic alterations make these animals a suitable model of absence epilepsy and epileptogenesis. An initial latent phase ends at the appearance of the first spontaneous seizure at about 2–3 months after WAG/Rij birth (Russo E. et al., 2016). The present results show that the pain threshold alterations are already present at the time of epilepsy onset and, in contrast to the aggravation of epilepsy, these pain alterations are not modified over aging having all values stable over time in the age window of 2–8 months as demonstrated by our results. Mechanical and thermal hyperalgesia (the increased sensitivity to a noxious stimulus) and mechanical allodynia (the painful response to a non-noxious stimulus) were significantly higher in WAG/Rij rats in comparison to Wistar at 2 months of age; this was steadily maintained on months 4 and 7 (respectively, three and six of treatment). These data agree with Velioglu et al. (2017) that observed an increased response to a hot source (Plantar test) in 2- and 8-months-old WAG/Rij rats; furthermore, neuroanatomical evidence suggests common circuits shared from generalized absence epilepsy and nociception (Schnitzler and Ploner, 2000; Velioglu et al., 2017).

BUT repeated administration normalized pain threshold alterations of WAG/Rij rats after 1, 3, and 6 months of treatment without clear signs of tolerance development. BUT efficacy was shown in response to non-noxious and noxious stimuli as well as mechanical and thermal stimuli. Moreover, a potentiation was obtained when BUT and VPA were coadministered at least in the relief of thermal hypersensitivity. On the contrary, VPA alone was ineffective with the exception of a mild but significant effect on month 1 of treatment in the Randall–Selitto test. The efficacy of BUT and BUT + VPA was lost 30 days after treatment discontinuation; notably, when administration was restarted after this latter suspension, 8 days were enough to reach again the full activity confirming the lack of tolerance development.

Based on our previous published data (Citraro et al., 2020), we may hypothesize that HDAC inhibition reaches a ceiling effect regarding its pain relieving potential and, therefore, adding VPA to BUT or *vice versa*, even though it increases HDAC inhibition, does not evoke an additive effect similarly to what we have observed on antiabsence effects. On the other hand, while both VPA and BUT decrease absence seizures development with this protocol, only BUT has important pain relieving effects (Citraro et al., 2020). This indicates that reducing absence seizures is not enough to modify pain in this strain as it was previously demonstrated for depressive-like behavior and cognitive impairment (Russo et al., 2011; Leo et al., 2019). An intriguing possibility would be that the increased allodynia along with all other pain-related alterations observed may be linked to ileal inflammation observed in this rat strain (personal not yet published data recently presented in a poster at the 39th° Congress of Italian Pharmacological Society available at http://www.congresslife.com/e-poster/sif2019/def/P330.pdf). In fact, we previously demonstrated that BUT but not VPA influences seizure susceptibility also by its intestinal anti-inflammatory effects (De Caro et al., 2019); therefore, this latter mechanism may contribute to the dual effect on both pain and seizures of BUT. However, this hypothesis needs to be tested.

The pain-relieving effect of the association BUT + VPA was pharmacodynamically studied using selective antagonists for main pain signaling regulators receptors. BUT + VPA seems to be able to orchestrate a complex antinociceptive response involving opioid, GABA, and PPAR-γ systems. The relevance of the three components appeared to be different when hyperalgesia and allodynia to different stimuli were analyzed, suggesting the possibility that diverse pain inhibitors can be recruited as response to the stimulation of different nociceptive pathways. The phenomenon may be originated by the epigenetic mechanism of BUT involving class I and II histone deacetylases (HDAC) through their inhibition (Berni Canani et al., 2012). HDAC regulates gene transcription through the modification of the chromatin structure by proteins acetylation, including not only histone proteins but also transcription factors (Russo R. et al., 2016). On this base, BUT inhibits nuclear factor κB (NFκB) activation, interferon γ production, and the upregulation of PPARγ (Berni Canani et al., 2012; Lee et al., 2017). All these mechanisms may cooperate to pain relief. NF-κB is a ubiquitously expressed protein complex regulating the transcription of genes involved in pain and inflammation. In the cytoplasm, NFkB is bound to inhibitors of κB (IκBα), upon activation the IκB kinase phosphorylates IκBα, and as a result p65/p50 complex is released and translocated to the nucleus where it initiates transcription of κB-associated inflammatory genes such as cyclooxogenase-2 (COX-2), IL-1β, and TNF-α that influence pain directly or indirectly (Hartung et al., 2015). Increased NFkB activity in immune and nervous system cells is associated to several chronic pain conditions in humans as well as pain in animals evoked by inflammation and nerve injury (Hartung et al., 2015). As regards PPARγ, alterations of this receptor are implied in glial cells dysfunctioning related to painful conditions (Di Cesare Mannelli et al., 2014); PPARγ agonists as well prevent neuropathic pain (Zanardelli et al., 2014). Russo R. et al. (2016) individuate the positive modulation of PPAR-α and -γ as a pivotal mechanism of BUT antinociceptive effects associated with the reduction of inflammatory markers (TNF-α, COX-2, iNOS, and cFOS) (Russo R. et al., 2016).

Furthermore, the block of HDAC with nonspecific inhibitors like BUT promotes nerve plasticity orientating neurons toward a neuroprotective status and promoting dendritic spines organization. HDAC inhibitors enhance synaptic plasticity and memory facilitating long-term memory formation (Stefanko et al., 2009). Therefore, histone alteration is crucial for regulating neurobiological processes such as neural network functions, synaptic plasticity, and synaptogenesis which also contribute to the pathophysiology of epilepsy. It is becoming evident that epigenetic regulatory mechanisms may also play a major role in epilepsy; modulation of chromatin structure through histone modifications has emerged as an important regulator of gene transcription in the brain. Altered histone acetylation seems to contribute to modification in gene expression associated with the epileptogenic process and epilepsy (Citraro et al., 2020). As recently described, BUT was able to reduce the development of absence epilepsy showing antiepileptogenic effects in WAG/Rij rats (Citraro et al., 2020) suggesting the relevant role of BUT for treating at the same time epilepsy phenomenology including pain. In WAG/Rij rats, also the other HDAC inhibitor VPA showed antiepileptogenic effects and the brain histone acetylation significantly increased during treatment with VPA or BUT alone and more strongly during coadministration (Citraro et al., 2020). VPA represents one of the most efficient antiepileptic drugs; it is used in human as an anticonvulsant and as a mood stabilizer. The pre- and post-synaptic effects of VPA depend on a very broad spectrum of actions, including the regulation of ionic currents and facilitation of GABAergic over glutamatergic transmission, and it displays HDAC inhibiting activity exerting a suppressive effect on gene transcription (Gottlicher et al., 2001). Corroborating the evidence of common biochemical and pathophysiological mechanisms in epilepsy and pain, VPA can reduce different types of persistent pain including neuropathic (Gill et al., 2011). Nevertheless, VPA was only partially able to relieve painful hypersensitivity in WAG/Rij rats suggesting the peculiarity of epilepsy-related pain threshold alterations distinct from neuropathic pain. This partial effect could be justified by the lower efficacy of this dose of VPA on HDAC in comparison to BUT; however, a higher dose cannot be used considering VPA toxicity and the unavoidable participation of other mechanisms (Citraro et al., 2020).

Although VPA, like BUT, shows analgesic and anti-inflammatory properties, it induces also detrimental effects including gastrointestinal disturbances, oxidative stress, renal and liver damage, neurological impairments, teratogenicity, and thrombocytopenia. Several authors reported the implication of an increased generation of free radicals and oxidative stress in neurotoxicity mechanisms of VPA (Zhang et al., 2010). Due to the close relationship between redox unbalance and (Chaudhary and Parvez, 2018) persistent pain (Di Cesare Mannelli et al., 2012), the oxidative state of the spinal cord of WAG/Rij rats after the different treatments was studied. VPA-treated rats showed higher concentration of carbonylated proteins in comparison to control. This is due to an oxidative modification of proteins induced by reactive oxygen species and other high reactive molecules such as hydroxynonenal; as a consequence carbonyl groups are introduced into protein side chains by a site-specific mechanism (Disatnik et al., 2000), inducing decreased functionality. Conversely, BUT and the combination BUT + VPA significantly prevented this alteration, suggesting a protection mediated by BUT on the pro-oxidative effects of VPA. Previously, a protective effect of BUT against protein carbonylation was demonstrated in different species (Song et al., 2010; Valvassori et al., 2016). Moreover, BUT was able to prevent liver toxicity in an animal model of steatosis (Raso et al., 2013) and we recently demonstrated that BUT also prevented VPA-induced hepatotoxicity (Pirozzi et al., 2020). In keep with its HDAC inhibitory activity, BUT has a strong impact on gene transcription upregulating mitochondrial and peroxisomal antioxidative genes and suppressing expression of several proinflammatory genes (Chriett et al., 2019). Accordingly, BUT (but also VPA and their combination) increased the glutathione reductase expression; this enzyme is a homodimer containing one FAD per monomer that catalyzes the reduction of glutathione disulphide to glutathione thus becoming essential for the glutathione redox cycle. Moreover, BUT reduced the expression of the inflammatory factor NFκB, a marker of nervous tissue dysregulation that resulted in increased hippocampus of patients with temporal lobe epilepsy (Teocchi et al., 2013). So higher protective properties of BUT in comparison to VPA could explain the difference in the regulation of pain threshold, and the coadministration of both is suggested as an integrated antiepileptic/pain relieving treatment with a possible better safety profile.

## Conclusion

In conclusion, WAG/Rij rats develop a persistent pain characterized by hypersensitivity to mechanical and thermal noxious and non-noxious stimuli. These arise in concomitance with spontaneous seizures’ appearance indicating the existence of common mechanisms. Here we demonstrate that one of those is HDAC although it is clear that some other are present. Nevertheless, it appears that while both have a common onset at the age of 2 months, but thereafter the two phenomena are apparently not directly linked, even if this deserves confirmation by further experiments. Finally, a repeated treatment with BUT (alone or in coadministration with VPA) offers an efficacious approach for relieving pain. In combination with its antiepileptic properties, results of the present study make the four-carbon short-chain fatty acid an ideal candidate to improve the quality of life of epileptic patients.

## Data Availability Statement

The raw data supporting the conclusions of this article will be made available by the authors, without undue reservation.

## Ethics Statement

The animal study and the procedure reported here were reviewed and approved by the Institutional Committee on the Ethics of Animal Experiments (CVS) of the University of Naples Federico II and by Ministero della Salute (protocol no. 371/2017-PR, 20/02/2017).

## Author Contributions

Conceptualization: AC, CG, and GDS; Methodology: CDC, JB, and RC; Validation: ER; Formal Analysis: CDC, LDCM, and LM; Investigation: CDC, LM, and RC; Data Curation: CDC, JB, RC, and RR; Writing – Original Draft Preparation: LDCM, CDC, and RR; Writing – Review and Editing: ER, RC, and RR; Supervision: AC, CG, and GDS; Funding Acquisition; AC, CG, and GDS.

## Funding

This work was supported by the Italian Ministry of University and Research (MIUR), Protocol No 2015XSZ9A2. This work was partly supported by the Italian Ministry of Health, Grant No GR-2013- 02355028.

## Conflict of Interest

The authors declare that the research was conducted in the absence of any commercial or financial relationships that could be construed as a potential conflict of interest.
